# Case-Mix, Care Processes, and Outcomes in Medically-Ill Patients Receiving Mechanical Ventilation in a Low-Resource Setting from Southern India: A Prospective Clinical Case Series

**DOI:** 10.1371/journal.pone.0135336

**Published:** 2015-08-11

**Authors:** Balasubramanian Karthikeyan, Tamilarasu Kadhiravan, Surendran Deepanjali, Rathinam Palamalai Swaminathan

**Affiliations:** Department of Medicine, Jawaharlal Institute of Postgraduate Medical Education and Research, Dhanvantri Nagar, Puducherry, India; Azienda Ospedaliero-Universitaria Careggi, ITALY

## Abstract

**Background:**

Mechanical ventilation is a resource intensive organ support treatment, and historical studies from low-resource settings had reported a high mortality. We aimed to study the outcomes in patients receiving mechanical ventilation in a contemporary low-resource setting.

**Methods:**

We prospectively studied the characteristics and outcomes (disease-related, mechanical ventilation-related, and process of care-related) in 237 adults mechanically ventilated for a medical illness at a teaching hospital in southern India during February 2011 to August 2012. Vital status of patients discharged from hospital was ascertained on Day 90 or later.

**Results:**

Mean age of the patients was 40 ± 17 years; 140 (51%) were men. Poisoning and envenomation accounted for 98 (41%) of 237 admissions. In total, 87 (37%) patients died in-hospital; 16 (7%) died after discharge; 115 (49%) were alive at 90-day assessment; and 19 (8%) were lost to follow-up. Weaning was attempted in 171 (72%) patients; most patients (78 of 99 [79%]) failing the first attempt could be weaned off. Prolonged mechanical ventilation was required in 20 (8%) patients. Adherence to head-end elevation and deep vein thrombosis prophylaxis were 164 (69%) and 147 (62%) respectively. Risk of nosocomial infections particularly ventilator-associated pneumonia was high (57.2 per 1,000 ventilator-days). Higher APACHE II score quartiles (adjusted HR [95% CI] quartile 2, 2.65 [1.19–5.89]; quartile 3, 2.98 [1.24–7.15]; quartile 4, 5.78 [2.45–13.60]), and new-onset organ failure (2.98 [1.94–4.56]) were independently associated with the risk of death. Patients with poisoning had higher risk of reintubation (43% vs. 20%; *P* = 0.001) and ventilator-associated pneumonia (75% vs. 53%; *P* = 0.001). But, their mortality was significantly lower compared to the rest (24% vs. 44%; *P* = 0.002).

**Conclusions:**

The case-mix considerably differs from other settings. Mortality in this low-resource setting is similar to high-resource settings. But, further improvements in care processes and prevention of nosocomial infections are required.

## Introduction

Mechanical ventilation is an important organ support treatment given to patients admitted in intensive care units (ICUs). Apart from requiring specialised equipment and logistics, trained healthcare personnel are also needed to provide care to mechanically ventilated patients. Availability of all these elements is essential for effective care delivery. But, there is a marked global disparity in healthcare resources (material, human, and economic) available to deliver ICU care including mechanical ventilation [1,2,3]. Hence, mechanical ventilation is sometimes viewed as a healthcare intervention with high opportunity costs in the developing and underdeveloped countries [4,5].

Apart from disparities in resources, the pattern of critical illnesses encountered in such settings is likely to substantially differ from the developed world. Therefore, it is essential to know whether the use of mechanical ventilation in low-resource settings is clinically effective. The outcomes of mechanical ventilation have been characterised in large unselected multinational patient populations from the developed world [6]. These studies suggest that outcome is influenced by the baseline characteristics, emergent clinical events, as well as factors related to patient management. On the other hand, despite the availability of mechanical ventilation services in the public- and private-run healthcare facilities in low-resource settings for more than three decades now, very few studies have systematically looked at the outcomes in unselected patient groups receiving mechanical ventilation in these settings. To address this gap, we conducted the present study looking at the characteristics, care practices, 90-day survival, and factors influencing the latter in adults receiving mechanical ventilation for non-surgical illnesses at a teaching hospital in southern India.

## Materials and Methods

### Study design and setting

This is a prospective hospital-based case series study of eligible patients receiving mechanical ventilation. The study protocol was reviewed and approved by the Institute Ethics Committee (Human studies) at Jawaharlal Institute of Postgraduate Medical Education and Research (JIPMER), Puducherry (Approval No. SEC/2011/1/1). The study was conducted at the Medical ICU of JIPMER Hospital during the period February 2011 to August 2012. This is a government-run teaching hospital located in the southern Indian town of Puducherry providing free-of-charge primary as well as referral care services to patients from the union territory of Puducherry and surrounding regions of the state of Tamil Nadu. In the Medical ICU, we take care of critically-ill non-surgical patients aged more than 12 years who are transferred from the emergency department and hospital floors. This is an eight-bedded ICU manned exclusively by the Department of Medicine. During the study, this ICU switched from an ‘open’ to ‘closed’ pattern of care led by a full-time internal medicine consultant during the daytime since July 2011. Postgraduate residents in their second and third years of residency program are posted in the ICU on 12-hour work shifts for a total duration of 2–3 months. The nurse-to-patient ratio of this ICU is 1:4.

### Participants

Patients were included in the study after obtaining informed written consent from the next of kin. Patients were eligible for inclusion if they were aged more than 12 years, had a medical-illness as the cause of hospitalization, and were receiving mechanical ventilation. Patients were excluded if they had any one of the following—patients transferred intubated from another hospital; patients receiving mechanical ventilation for < 24 hours; baseline evaluation could not be performed in the first 24 hours following intubation; or patients on non-invasive ventilation.

### Baseline evaluation

The following parameters were recorded at the baseline evaluation—age, gender, smoking status, alcohol abuse, primary diagnosis, severity of illness scores (acute physiology and chronic health evaluation II [APACHE II] and simplified acute physiology score II [SAPS II]), and presence of co-morbidities such as diabetes, hypertension, and asthma. Laboratory parameters including blood counts, serum creatinine, electrolytes, liver function tests, and arterial blood gas analysis were also noted at this evaluation. Presence of organ failure at baseline was defined using the sequential organ failure assessment (SOFA) definitions [7]—patients were considered to have respiratory failure if PaO_2_/FiO_2_ was ≤ 300; hematologic failure if platelet count was ≤ 100,000/μL (≤ 100×10^9^/L); hepatic failure if bilirubin was ≥ 2 mg/dL (≥ 34.2 μmol/L); circulatory failure if they required any dose of vasopressor/inotrope; neurological failure if Glasgow coma score was < 13; and renal failure if the serum creatinine was ≥ 2 mg/dL (≥ 176.8 μmol/L).

### Outcomes and definitions

Patients were followed-up during hospital stay and the outcomes were assessed in three domains—disease-related, mechanical ventilation-related, and process of care-related outcomes. Disease-related outcomes assessed were in-hospital mortality, 90-day mortality, and new-onset organ failure. The same cut-offs as above were used to define new-onset organ failure. The 90-day survival was assessed through telephonic inquiry. Following hospital discharge, patients or their family members were contacted over the telephone on Day 90 (from the day of intubation) or later to assess the vital status of the patient. If the patient could not be contacted even after multiple attempts, then the patient was adjudicated as lost to follow-up.

Mechanical ventilation-related outcomes studied were duration of ventilation, timing of tracheostomy, barotrauma, endotracheal tube block, unplanned extubation, weaning failure, and extubation failure. Prolonged mechanical ventilation was defined as ventilation for > 21 days [8]. Weaning outcome was classified into three groups namely simple, difficult, and prolonged weaning. Simple weaning was defined as successful extubation following the first spontaneous breathing trial; difficult weaning as success in two or three attempts; and prolonged weaning as more than three attempts [9]. Barotrauma was defined as radiographically confirmed interstitial emphysema, pneumothorax, pneumomediastinum, pneumoperitoneum, or subcutaneous emphysema that could not be attributed to iatrogenic injury. Endotracheal tube block was defined as partial or complete blockade of the endotracheal tube on visual inspection after a planned/unplanned extubation. Any self-extubation by the patient or accidental extubation during position change or patient movement, or extubation for a blocked tube was considered as unplanned extubation.

Process of care-related outcomes studied were deep vein thrombosis (DVT), pressure sores, nosocomial infections, and adherence to standard of care practices (DVT prophylaxis, stress ulcer prophylaxis, and head-end elevation). In addition to DVT confirmed by compression ultrasound, clinically suspected but unproven episodes were also considered as incident DVT. Ventilator-associated pneumonia (VAP) was defined as both confirmed as well as suspected episodes of VAP as diagnosed by the treating physician that necessitated treatment with antibiotics.

### Data sources and bias

Data on in-hospital mortality and new-onset organ failure were prospectively collected from case records. We anticipated that data for some outcomes such as endotracheal tube block and unplanned extubation might not be reliably captured in case records. Hence, in addition to perusing case records, the care providers were also interviewed to obtain this information. Documentation of nosocomial infections in case records could suffer from underreporting bias for various reasons. To avoid this, we used much broader criteria to adjudicate on VAP—while this is expected to be very sensitive, its specificity for VAP diagnosis is likely to be modest. Adherence to head-end elevation and presence of pressure sores were assessed by the investigator during data collection visits. All data were collected by the same investigator (BK) ensuring uniformity.

### Sample size calculation

Since published estimates of 90-day mortality from a similar setting were unavailable to inform the sample size calculation, we assumed the 90-day mortality to be 50%. To estimate this within 7 percentage points of the true value (absolute precision) with 95% confidence, we needed to study 196 patients [10]. Allowing for a 20% drop-out rate, we calculated the minimum required sample size as 235 patients.

### Statistical analyses

Continuous variables with an approximate normal distribution are summarized as mean ± SD or as median (interquartile range, IQR) when the distribution is skewed. Categorical variables are presented as frequency with proportions, n (%). We present 95% confidence intervals for point estimates of mortality, and describe the risk of nosocomial infections as incidence-density. We used the Kaplan-Meier survival plot to depict the survival distribution of patients, and compared the survival curves between groups by log-rank test. While analysing risk factors for mortality, we excluded patients that were lost to follow-up and that left care against medical advice. We compared the continuous variables between survivors vs. non-survivors by independent sample t-test or Wilcoxon rank-sum test as appropriate, and by chi-square test for categorical variables. We assessed the impact of case-mix by comparing the subgroup of acute poisoning patients with the rest.

We performed a multivariable analysis by Cox proportional hazards regression to adjust for confounding. Variables associated with 90-day mortality at *P* < 0.1 threshold on univariate analysis were considered for entry into the model. To avoid overfitting, we built a parsimonious model with the least number of explanatory variables [11]. Hence, we did not include redundant variables, whose information was already contained in other composite variables, despite crossing the *P* < 0.1 threshold. APACHE II score was entered as an indicator variable after dividing the study sample into four nearly equal-sized groups based on quartiles of APACHE II score. Other quantitative variables such as age and serum albumin were entered as such without dichotomizing. A statistical software package (Stata/IC for Windows, version 12.1, Stata Corp., College Station, Texas) was used for analysis. All *P*-values were two-sided, and a *P* < 0.05 was considered statistically significant.

## Results

**Fig 1:**
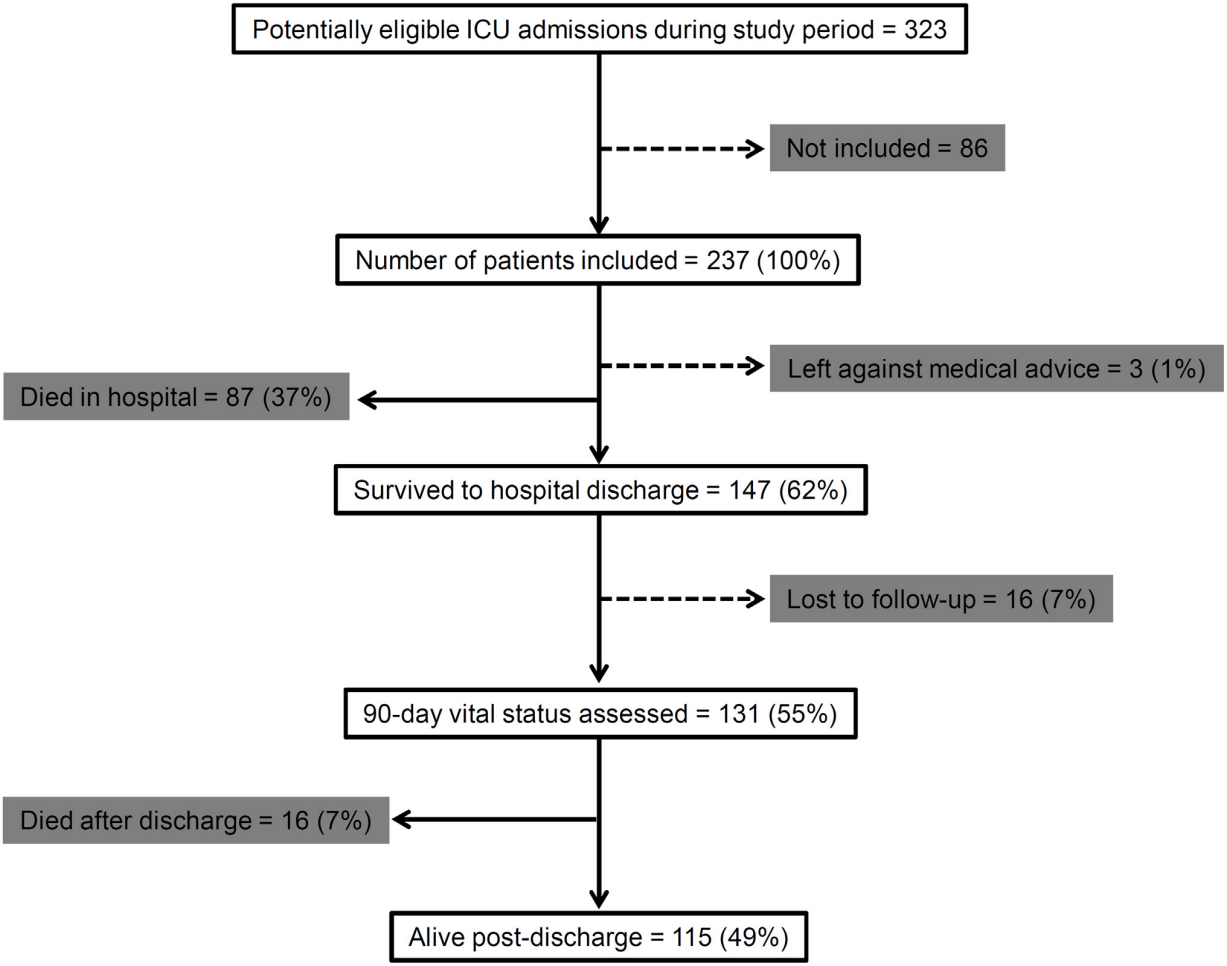
Schematic showing the flow of patients through the study.

Of the 323 potentially eligible patients admitted to the Medical ICU during the study period, we studied 237 patients satisfying the inclusion/exclusion criteria. The follow-up was censored on August 31, 2012. Of the 237 patients, 16 (7%) were lost to follow-up after hospital discharge; 3 (1%) patients left care against medical advice (Fig 1). Two patients had completed only 70 days and 83 days of follow-up at study closure, and their follow-up was censored at that point.

### Demographics and case-mix

The mean age of the patients was 40 ± 17 years and 140 (51%) were men. The baseline characteristics are presented in Table 1. Diabetes was a common co-morbidity present in 38 (16%) patients. Poisoning and envenomation accounted for 98 (41%) of 237 admissions; the most common poisoning encountered was insecticide poisoning (79 [95%] of 83 patients). Unclassified indications for ICU admission included hanging (11 patients), tetanus (6 patients), renal failure (6 patients), autoimmune diseases (5 patients), hypokalemic paralysis (2 patients), acute myeloid leukemia, and hepatic encephalopathy (1 patient each).

**Table 1:** Baseline characteristics of 237 adults receiving mechanical ventilation.

Characteristic	Frequency, n (%)
*Age-group*	
13–20 years	30 (13%)
21–30 years	61 (26%)
31–40 years	42 (18%)
41–50 years	34 (14%)
51–60 years	39 (17%)
61–70 years	21 (9%)
71 years and above	10 (4%)
Smoking	43 (18%)
Alcohol abuse	36 (15%)
*Co-morbid illnesses (not mutually exclusive)*	
Diabetes mellitus	38 (16%)
Hypertension	25 (10%)
COPD/asthma/tuberculosis	9 (4%)
*Indication for hospitalization*	
Acute poisoning	83 (35%)
Respiratory diseases (incl. infectious)	38 (16%)
Sepsis	30 (13%)
Neurologic diseases	30 (13%)
Envenomation	15 (6%)
Cardiovascular diseases	9 (4%)
Others*	32 (13%)
*Indication for mechanical ventilation*	
Impending respiratory failure	92 (39%)
Type 1 respiratory failure	91 (38%)
Type 2 respiratory failure	43 (18%)
Airway protection	11 (5%)
*Organ failure at baseline (not mutually exclusive)* ^†^	
Any organ failure (one or more)	164 (69%)
Respiratory failure	154 (65%)
Renal failure	62 (26%)
Neurological failure	58 (24%)
Circulatory failure	43 (18%)
Hematological failure	40 (17%)
Liver failure	16 (7%)

COPD = Chronic obstructive pulmonary disease

*See text for break-up;

^†^Defined by cut-offs corresponding to a sequential organ failure assessment (SOFA) score point of 2

### Organ failure

Respiratory failure was the most common organ failure at baseline, present in about two-thirds of patients; renal failure and neurological failure were present in a quarter of patients each; and nearly one-fifth of patients had circulatory failure at baseline (Table 1). The mean APACHE II score was 15.7 ± 8.5, and the mean SAPS II score was 35.0 ± 15.6. The cut-offs for APACHE II quartiles were score 1–9 (quartile 1; 62 patients), 10–15 (quartile 2; 63 patients), 16–21 (quartile 3; 55 patients), and > 21 (quartile 4; 57 patients). During ICU stay, 89 (38%) patients developed one or more new-onset organ failures. The most common new-onset organ failure was circulatory failure (45 [19%] patients); renal failure (21 [9%]), respiratory failure (18 [8%]), neurological failure (18 [8%]), hematological failure (10 [4%]), and liver failure (5 [2%]) were the other new-onset organ failures.

### Mechanical ventilation-related outcomes

Volume-cycled assist-control mandatory ventilation (ACMV) was the initial mode in all patients. The mean tidal volume used was 389 ± 42 mL. The mean plateau pressure and mean PEEP were 18.2 ± 2.0 cm H_2_O and 5.0 ± 2.2 cm H_2_O, respectively. The maximum FiO_2_ used was 64.6 ± 21.2%. The median duration of mechanical ventilation was 8 (5–14) days. Mechanical ventilation related outcomes are presented in Table 2. In total, 20 patients needed prolonged ventilation– 18 of them had failed weaning; the other two had tetanus. Seven of them died in hospital; three died after discharge; and three were lost to follow-up. The median time to tracheostomy was 11 (9–14) days. Of the nine (4%) patients that developed barotrauma, eight had pneumothorax and the other had subcutaneous emphysema. While most patients had an endotracheal tube block only once (80 patients), some patients had more than one instance of blockade—8 patients had twice and 3 patients had thrice. Of the 47 (20%) patients that had unplanned extubation, 37 had one unplanned extubation, and 8 had two unplanned extubations; 18 unplanned extubations were attributable to tube blockade.

**Table 2:** Mechanical ventilation-related outcomes in 237 ICU patients.

Outcome	Frequency, n (%)
Prolonged mechanical ventilation*	20 (8%)
Tracheostomy	41 (17%)
Barotrauma	9 (4%)
Unplanned extubation	47 (20%)
Endotracheal tube block	91 (38%)
Failed first weaning attempt^†^	99/171 (58%)
Re-intubation after planned extubation^†^	50/171 (29%)

*Defined as mechanical ventilation > 21 days;

^†^Denominator is the number of patients in whom weaning was attempted

Weaning was attempted in 171 (72%) patients– 159 were given spontaneous breathing trial through a T-piece; in 10 patients other methods were used; and data was missing in 2 patients. Of the 171 patients, 71 (42%) had a simple weaning in the first attempt; 81 (47%) had difficult weaning; 18 (11%) had prolonged weaning; and weaning outcome was missing in 1 patient. Most patients failing the first attempt (78 of 99 [79%]) could be weaned off and discharged from hospital; 10 of them needed prolonged ventilation beyond Day 21. The remaining 21 patients failing the first weaning attempt died in the hospital (20 patients) or left against medical advice (1 patient); 8 of these 21 patients required prolonged mechanical ventilation (S1 Fig). Fifty (29%) patients needed reintubation following a planned extubation. Such extubation failure occurred only once in 43 of the 50 patients; twice in 5 patients; and 2 patients failed extubation thrice.

### Process of care-related outcomes

**Fig 2:**
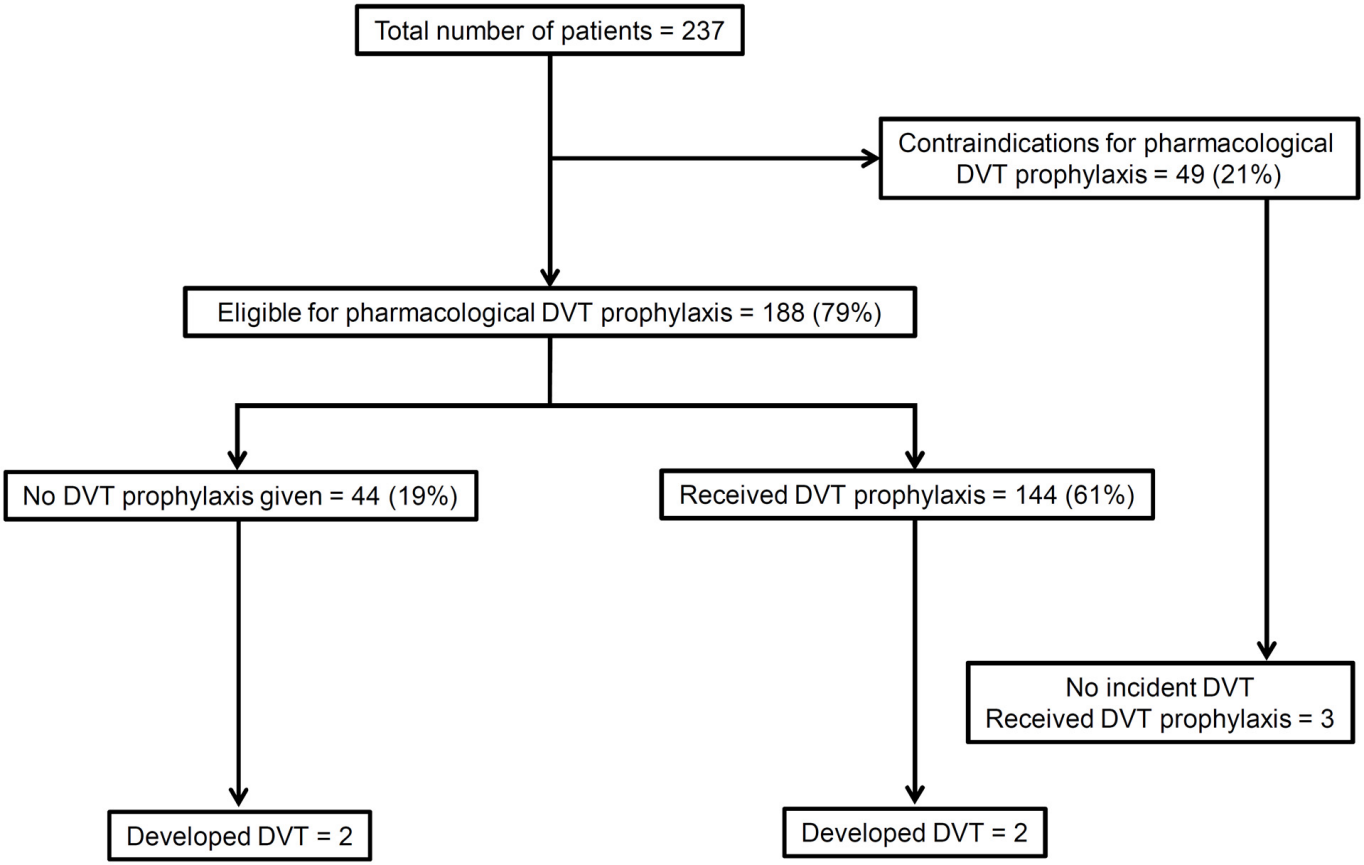
Adherence to deep vein thrombosis (DVT) prophylaxis and incident DVT in 237 adults receiving mechanical ventilation.

One hundred and forty seven (62%) patients received DVT prophylaxis—enoxaparin 135 (57%); unfractionated heparin 11 (5%); and elastic compression stockings 1 patient. Forty-nine (21%) patients had contraindications for pharmacological prophylaxis. In total, four (2%) patients developed lower limb DVT (Fig 2)—two had clinically suspected DVT and in the other two it was confirmed by compression ultrasound. One patient developed symptomatic non-fatal pulmonary thromboembolism despite DVT prophylaxis. Overall, 212 (89%) patients received stress ulcer prophylaxis with either proton pump inhibitors or H2 receptor blockers. Head-end elevation was implemented in 164 (69%) patients. Pressure sores developed in 67 (28%) patients. Half the patients (116 [49%]) had a central venous catheter. All patients had a Foley’s catheter for bladder drainage and a nasogastric tube for enteral feeding during the period of mechanical ventilation. We did not collect data on use of sedation, neuromuscular blockers, and nutritional support.

In total, 147 (62%) patients developed at least one nosocomial infection. The details of nosocomial infections are presented in Table 3. The median time to development of VAP was 4 (3–6) days.

**Table 3:** Incidence of nosocomial infections in 237 adults receiving mechanical ventilation.

Nosocomial infection	Frequency, n (%)	Incidence density	Culture-positivity rate
At least one nosocomial infection	147 (62%)	58.4 per 1,000 patient-days	—
Ventilator-associated pneumonia	144 (61%)	57.2 per 1,000 ventilator-days	108/144
Catheter-associated urinary tract infection	28 (12%)	11.1 per 1,000 catheter-days	26/28
Central line associated bloodstream infection^†^	12/116 (10%)	9.2 per 1,000 catheter-days	11/12*
Skin and soft tissue infection	5 (2%)	2.0 per 1,000 patient-days	5/5

*In one patient blood culture was sterile; but, the catheter tip culture yielded growth;

^†^Denominator is the number of patients with a central venous catheter

### In-hospital and 90-day mortality

Of the 237 patients, 87 (37% [95% CI 31%- 43%]) died in the hospital; 147 (62% [56%- 68%]) survived to hospital discharge. The median duration of follow-up for the survivors was 150 (101–203) days. Sixteen patients (7% [4%-11%]) died at home during the follow-up period; and 115 (49% [42%- 55%]) patients were alive on Day 90 or later (Fig 1). Details of patients that died during follow-up are presented in S1 Table. The median time to death in these patients was 35 (25–82) days since intubation. Eight of them had been discharged with a tracheostomy tube in situ.

**Fig 3:**
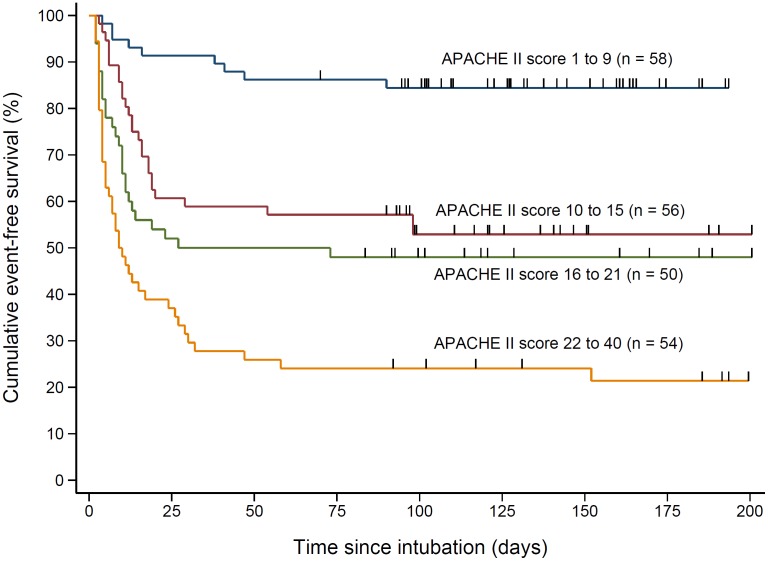
Kaplan-Meier survival plots of patients stratified by the quartile of APACHE II score. Hash marks on the plot indicate censoring. In two patients follow-up was censored at 70 days and 83 days.

**Fig 4:**
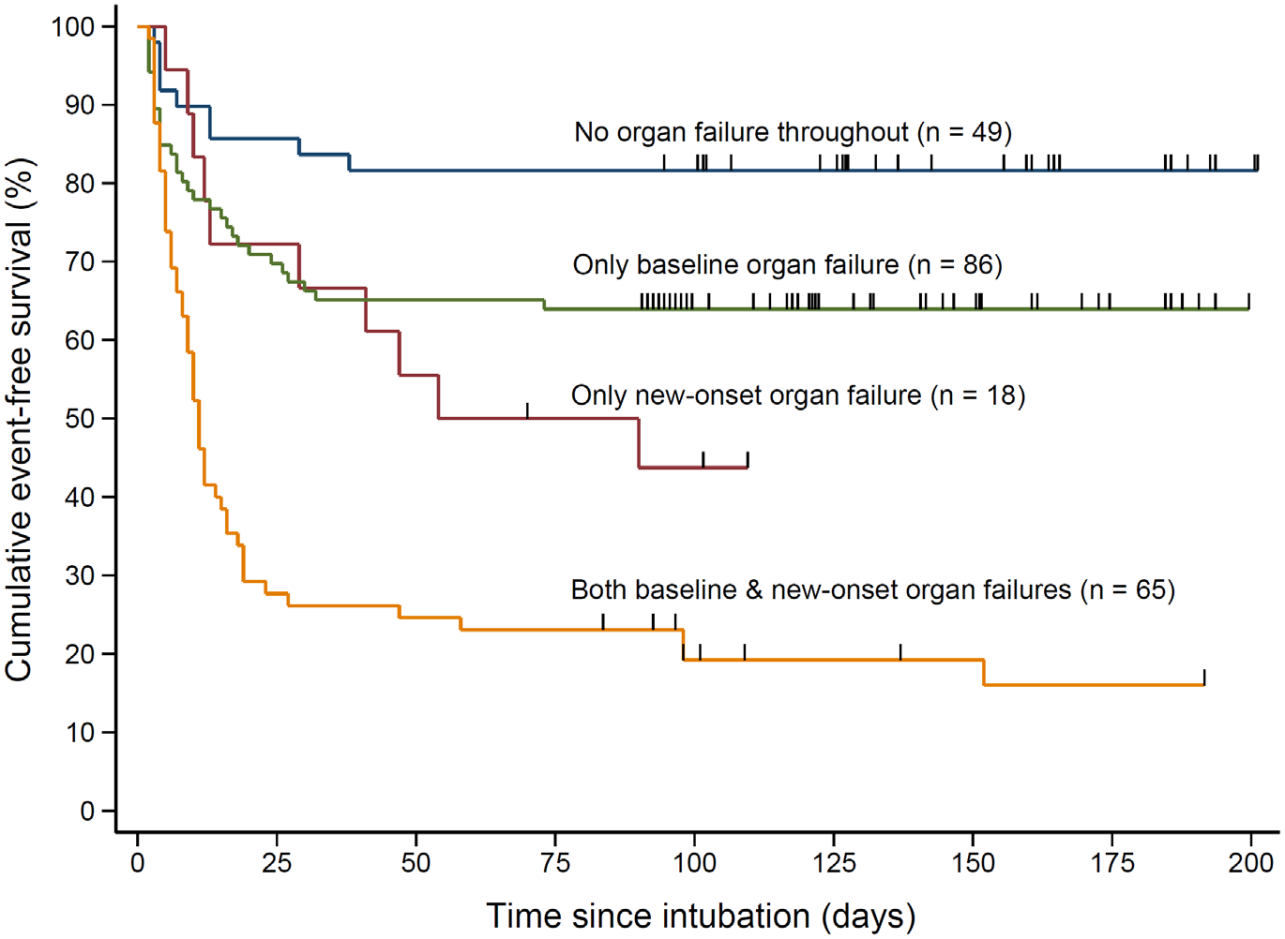
Cumulative impact of baseline and/or new-onset organ failures on 90-day survival. Hash marks on the plot indicate censoring. In two patients follow-up was censored at 70 days and 83 days.

The mean APACHE II score of patients that died was significantly higher compared to the survivors (20.1 ± 8.5 vs. 11.7 ± 6.9; *P* < 0.001). There was a progressive increase in the risk of mortality in the upper APACHE II quartiles compared to the lowest quartile (Fig 3). The risk of death was about 2.5-fold in patients with one or more organ failure at baseline compared to those without any organ failure (S2 Fig; unadjusted hazard ratio = 2.52 [1.53–4.16]; *P* < 0.001). Likewise, patients with new-onset organ failure had about 3.5-fold risk of mortality (S3 Fig; unadjusted HR = 3.55 [2.38–5.30]; *P* < 0.001). When cross-classified, patients with new-onset organ failure in addition to other organ failure at baseline had the least chances of survival; whereas, patients without any organ failure throughout had the best chances of survival (Fig 4).

Apart from these factors, age, serum albumin, diabetes, and hypertension were also associated with mortality on univariate analysis (Table 4). After adjusting for confounding, only higher APACHE II quartiles and new-onset organ failure were independently associated with the risk of death (Table 4). Other variables that were associated with mortality on univariate analysis, but not included in the multivariable model were (See: Statistical analyses)–higher SAPS II score (28.3 ± 11.7 vs. 42.1 ± 16.6; *P* < 0.001), longer duration of mechanical ventilation (8 [5–14] vs. 9 [4–14] days; *P* = 0.008), higher PEEP (median [range] 5 [0–10] vs. 5 [0–14] cmH_2_O; *P* = 0.033) and FiO_2_ requirements (57.7 ± 17.9% vs. 74.3 ± 22.1%; *P* < 0.001), higher plateau pressure (17.8 ± 1.8 vs. 18.6 ± 2.2 cmH_2_O; *P* = 0.007), lower platelet count (205 ± 125 vs. 169 ± 118 × 10^3^/μL; *P* = 0.026), and lower hematocrit (37.8 ± 6.4% vs. 33.2 ± 6.9%; *P* < 0.001).

**Table 4:** Risk factors for 90-day mortality in mechanically ventilated ICU patients.

Explanatory variable	Survivors (n = 115)	Dead (n = 103)	Unadjusted HR (95% CI)	*P*-value	Adjusted HR (95% CI)*	Adjusted *P*-value*
Age (years)	36.0 ± 16.1	44.2 ± 17.9	1.02 (1.01–1.03)	0.001	1.01 (1.00–1.02)	0.156
APACHE II score						
Quartile 1	49/58 (84%)	9/58 (16%)	1.00^†^	—^†^	1.00^†^	—^†^
Quartile 2	30/56 (54%)	26/56 (46%)	3.58 (1.68–7.65)	0.001	2.65 (1.19–5.89)	0.017
Quartile 3	24/50 (48%)	26/50 (52%)	4.68 (2.19–9.99)	< 0.001	2.98 (1.24–7.15)	0.014
Quartile 4	12/54 (22%)	42/54 (78%)	9.04 (4.39–18.64)	< 0.001	5.78 (2.45–13.60)	< 0.001
Organ failure—baseline	67 (58%)	84 (81%)	2.52 (1.53–4.16)	< 0.001	1.13 (0.62–2.05)	0.688
Organ failure—new-onset	20 (17%)	63 (61%)	3.55 (2.38–5.30)	< 0.001	2.98 (1.94–4.56)	< 0.001
Serum albumin (g/dL)	3.2 ± 0.5	2.8 ± 0.6	0.45 (0.33–0.61)	< 0.001	0.82 (0.57–1.19)	0.303
Diabetes	12 (10%)	25 (24%)	1.81 (1.15–2.85)	0.010	0.78 (0.46–1.33)	0.365
Hypertension	7 (6%)	18 (17%)	1.89 (1.13–3.14)	0.015	0.98 (0.52–1.87)	0.956

APACHE II = Acute physiology and chronic health evaluation II

*Complete data set for multivariable analysis was available for 214 patients including 101 deaths;

^†^Referent quartile

### Influence of case-mix on outcomes

The subgroup of patients with acute poisoning was significantly younger and more likely to be male (Table 5). Co-morbidities such as diabetes and hypertension were less common. They had better APACHE II and SAPS II scores, and lower frequency of baseline as well as new-onset organ failures. They had a significantly higher risk of VAP and reintubation. Notwithstanding, their in-hospital and 90-day survival was significantly better than the rest (Table 5).

**Table 5:** Comparison of characteristics and outcomes between patients with poisoning and the rest.

Characteristic/Outcome	Acute poisoning group (n = 83)	Other diagnostic groups (n = 154)	*P*-value
Age (years)	34.3 ± 14.5	42.6 ± 18.0	< 0.001
Male gender	60 (72%)	80 (52%)	0.002
Diabetes	—	38 (25%)	< 0.001
Hypertension	3 (4%)	22 (14%)	0.011
Smoking	26 (31%)	17 (11%)	< 0.001
Alcohol abuse	22 (27%)	14 (9%)	< 0.001
APACHE II score	10.3 ± 6.8	18.6 ± 7.9	< 0.001
SAPS II score	25.1 ± 12.5	40.4 ± 14.5	< 0.001
Serum albumin (g/dL)	3.3 ± 0.5	2.9 ± 0.6	< 0.001
Organ failure—baseline	35 (42%)	129 (84%)	< 0.001
Organ failure—new-onset	25 (30%)	64 (42%)	0.083
Duration of ventilation (days)	9.9 ± 5.5	11.0 ± 9.5	0.270
Tracheostomy	10 (12%)	31 (20%)	0.117
Endotracheal tube blockade	37 (45%)	54 (35%)	0.151
Unplanned extubation	21 (25%)	26 (17%)	0.121
Reintubation*	29/67 (43%)	21/104 (20%)	0.001
VAP	62 (75%)	82 (53%)	0.001
Death (in-hospital)	20/83 (24%)	67/151 (44%)	0.002
Death (90-day)	20/78 (26%)	83/140 (59%)	< 0.001

APACHE II = Acute physiology and chronic health evaluation II; SAPS II = Simplified acute physiology score II; VAP = Ventilator-associated pneumonia

*Denominator is the number of patients in whom weaning was attempted

## Discussion

We found that the case-mix of patients receiving mechanical ventilation for medical-illness in our setting was dominated by acute poisoning, and this had a bearing on the clinical outcome in these patients. We also found that, notwithstanding the limited resources, the mortality was similar to that documented elsewhere. But, the care practices and outcomes miss the benchmark in some key areas such as adherence to ventilator bundle and risk of nosocomial infections. Further, this study also uncovered that a considerable proportion of patients die after hospital discharge.

The current study has several strengths—i) the sample size was large enough to obtain precise estimates of mortality and to examine the risk factors for 90-day mortality; ii) data collection was prospective; iii) the study sample was not restricted to any particular diagnostic group; iv) a holistic assessment of outcomes was done in three domains; and v) the loss to follow-up was minimal. On the other hand, the present study has some limitations. First, we did not attempt to verify the accuracy of a diagnosis of VAP. Notwithstanding, this metric reflects the real life situation, at least in our setting. Second, the follow-up period was comparatively short to assess post-discharge mortality. Nonetheless, to our knowledge, this is the first study from India to provide an empirical estimate of the post-discharge mortality. Third, we did not study the use of sedation, neuromuscular blockers, and nutritional support.

In the present study, poisoning and envenomation accounted for nearly 40% of ICU admissions requiring mechanical ventilation. This is strikingly different from developed countries where most of the admissions were due to pneumonia, chronic obstructive pulmonary disease, sepsis, heart failure, and neurological diseases [6,12]. This finding also differs from other developing countries such as Brazil [13]. The case-mix broadly reflects the relative abundance of poisoning, envenomation, and infectious diseases among patients presenting to the emergency services of our hospital. Further, our patients were younger compared to western countries (mean age 59–61 years) [6,12]. This finding is similar to earlier studies from India, where the mean age was found to be 41 and 43 years [14,15]. On the other hand, the SAPS II scores were lower in the present study compared to that by the Mechanical Ventilation International Study Group (MV-ISG) [12]. In our setting, only 5% of the intubations were for airway protection, which is considerably less compared to other settings where 17% were intubated for airway protection [6]. This possibly reflects the scarcity of ventilators in resource-limited settings. ACMV is the mode that is widely used as the initial ventilation mode. In the MV-ISG study, 66% of patients received ACMV as the initial mode of ventilation [6]. Interestingly, all patients in the present study were initiated on ACMV. This probably reflects a local variation in practice rather than any particular reason.

In the present study, the in-hospital mortality was 37%. Esteban et al found that the in-hospital mortality of mechanically ventilated patients had improved from 40% to 35% over the period of 1998 to 2010 in three multinational cohorts [12]. Thus, the in-hospital mortality in our setting is comparable to other countries. But, the present findings are in sharp contrast to historical studies from low-resource settings that had often documented mortality rates exceeding 70% [14,16,17]. In a contemporary study from Brazil, the in-hospital mortality of patients on invasive mechanical ventilation was 43%, quite similar to ours [13]. These observations suggest that mortality of mechanically ventilated patients has improved in low-resource settings also. It is known for long that many ICU patients die after hospital discharge [18]. In the United States, 30% of mechanically ventilated Medicare beneficiaries older than 65 years died within 6 months of hospital discharge [19]. Compared to this, the post-discharge mortality in our study was lower probably because our study population was much younger, and the follow-up was shorter.

The mean duration of mechanical ventilation in the present study was longer compared to the findings of the MV-ISG (10.6 vs. 5.9 days) [6]. The time taken for weaning was not included in the duration of mechanical ventilation by the MV-ISG. If included, then the duration would be similar (10.6 vs. 10.1 days). Among the mechanical ventilation-related outcomes, endotracheal tube block, unplanned extubation, and reintubation rates were particularly high in our study. But, most instances of tube blockade were incidentally discovered, the significance of which is unclear. Higher rate of tube block in our study might be due to the use of large doses of atropine in patients with insecticide poisoning. In earlier studies, the rate of unplanned extubations was between 11–14% [20,21]. Infrequent use of sedation, agitation due to atropine delirium, and inadequate staffing could account for a higher rate of unplanned extubations in our setting. The high reintubation rate was largely explained by differences in case-mix—reintubation was particularly common in patients with acute poisoning. This could be due to two reasons—i) VAP was more common among them; and ii) neuromuscular weakness due to intermediate syndrome might also contribute to some extent [22]. But, we did not study this aspect.

We found that most patients received DVT prophylaxis, and 17% patients did not receive DVT prophylaxis despite having no contraindication. In the multinational ENDORSE study, only 40% of at-risk medical patients received DVT prophylaxis [23]. The adherence was only 19% in the Indian centers of ENDORSE study [24]. Compared to these, the adherence was much better in the current study. Notwithstanding, all patients should have received some form of DVT prophylaxis as a part of ventilator bundle. Likewise, head end elevation was implemented in 69% of patients. Thus, the overall compliance with the ventilator bundle is only modest in our ICU setting, which needs improvement.

Risk of nosocomial sepsis, especially VAP, was high in our ICU. The incidence was very high compared to developed nations such as the United States and Scotland where it is 1.0 and 6.5 per 1,000 ventilator-days respectively [25,26]. However, a previous study by the International Nosocomial Infection Control Consortium from eight developing nations found a high incidence of VAP (24.1 per 1,000 ventilator-days) with a wide variation across different countries (10 to 53 per 1,000 ventilator-days) [27]. Likewise, a meta-analysis of studies from developing countries found a VAP rate of 22.1 per 1,000 ventilator-days [28]. Thus, the high burden of nosocomial infections is a problem common to many low-resource settings. Even then the infection rates were still higher in our setting. To some extent this could be attributed to systematic differences in the way we adjudicated on VAP. We suspect that a large number of such diagnoses were inaccurate. This suspicion is corroborated by the fact only 75% of VAP were culture-positive. Moreover, these were non-quantitative endotracheal aspirate cultures which lack specificity in differentiating colonization from true VAP [29]. Another potential reason for a high incidence of VAP could be that most intubations were crash intubations performed in the busy emergency room. Apart from obvious reasons such as overcrowding and poor hand hygiene, it remains to be explored whether the high environmental temperatures prevailing throughout the year in tropical climates add to the risk of nosocomial infections [30]. Despite a high incidence of nosocomial infections, we did not find an association with increased mortality. Although nosocomial infections increase the length of stay and cost of care, studies indicate that the attributable mortality of VAP in medical ICU patients is minimal [31]. Finally, we found that APACHE II score and new-onset organ failure were independently associated with 90-day mortality, which is well known [7,32]. But, organ failure at baseline was not independently associated with 90-day mortality, since information on organ failure at presentation is captured in the APACHE II score itself.

The present study indicates that mechanical ventilation could be clinically effective in low-resource settings. But, further improvements in care processes and prevention of nosocomial infections are necessary, and this calls for commitment of more resources to ICU services. The findings of the present study would be applicable to settings where a similar case-mix is encountered. Even when the case-mix differs, our findings might be applicable to low-resource settings with material resources for mechanical ventilation, but not specialist doctors, nurses, and other paramedical personnel. But, it is unclear whether the present findings could be generalised to non-teaching hospitals in low-resource settings.

## Conclusions

Acute poisoning dominates the case-mix of patients receiving mechanical ventilation in our setting. Characteristics of this subgroup of patients distinctly differ from the rest, and their prognosis is better. Moreover, the difference in case-mix explains at least partly the observed differences in outcomes such as reintubation and VAP. Finally, despite the resource limitations, the overall survival is similar to other settings. However, process of care measures such as adherence to ventilator bundle and prevention of nosocomial infections need concerted efforts for improvement.

## Supporting Information

S1 Fig Outcome of weaning in 237 mechanically ventilated patients. AMA = Left against medical advice; PMV = prolonged mechanical ventilation; SBT = Spontaneous breathing trial.
(TIF)

S2 Fig Kaplan-Meier survival plots of patients stratified by presence of organ failure at baseline. Hash marks on the plot indicate censoring. In two patients follow-up was censored at 70 days and 83 days.
(TIF)

S3 Fig Kaplan-Meier survival plots of patients stratified by new-onset organ failure. Hash marks on the plot indicate censoring. In two patients follow-up was censored at 70 days and 83 days.
(TIF)

S1 File Dataset. De-identified minimal dataset.
(XLS)

S1 Table Case details of mechanically ventilated patients that died after hospital discharge. APACHE II = Acute physiology and chronic health evaluation II.
(DOCX)
