# Supplementary figures and images for: Case-Mix, Care Processes, and Outcomes in Medically-Ill Patients Receiving Mechanical Ventilation in a Low-Resource Setting from Southern India: A Prospective Clinical Case Series
Source: PLoS One. 2015 Aug 11;10(8):e0135336. doi: 10.1371/journal.pone.0135336 (PMC4532502; doi:10.1371/journal.pone.0135336)

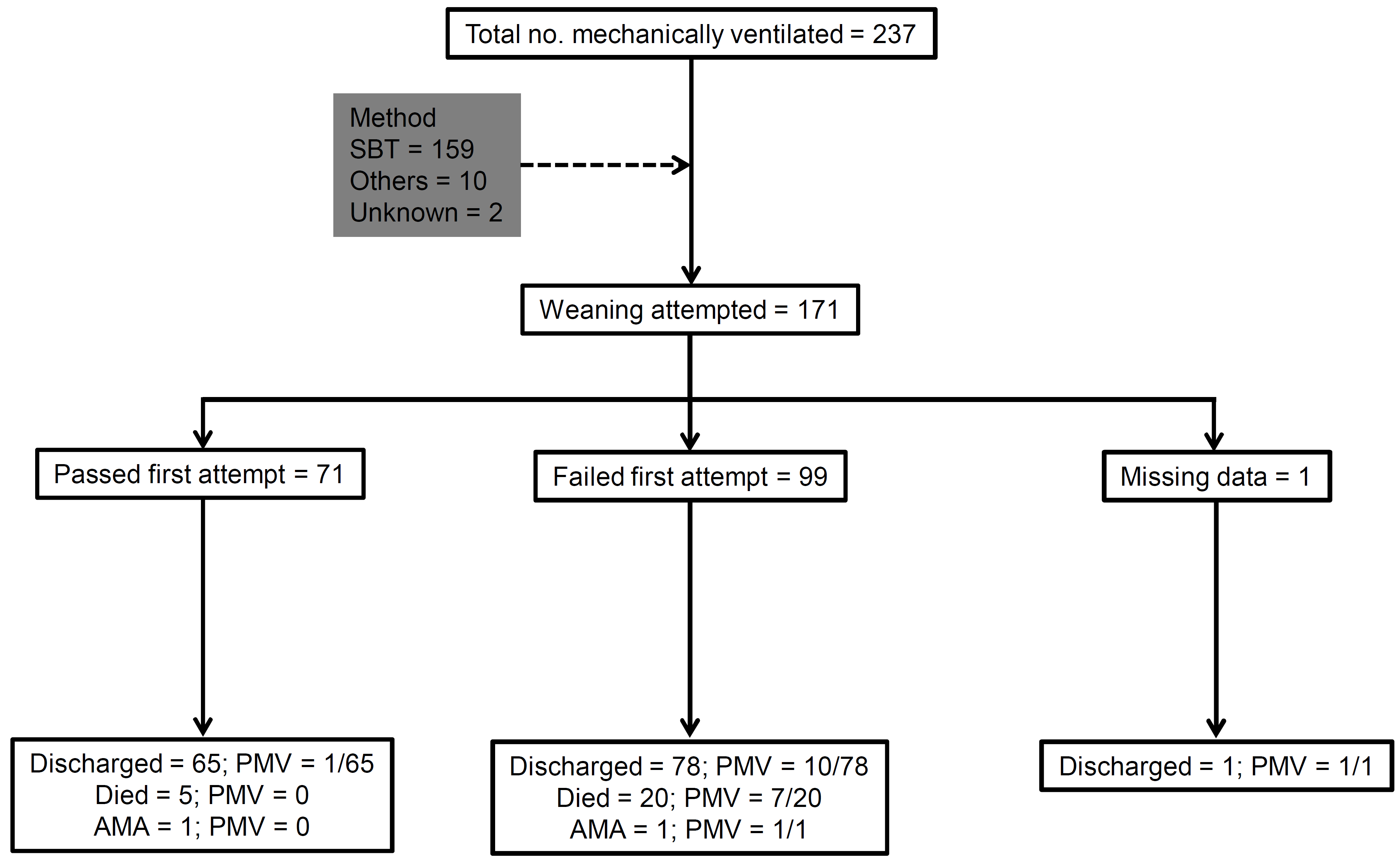

Supplement: S1 Fig — AMA = Left against medical advice; PMV = prolonged mechanical ventilation; SBT = Spontaneous breathing trial. (TIF) [file pone.0135336.s001.tif]

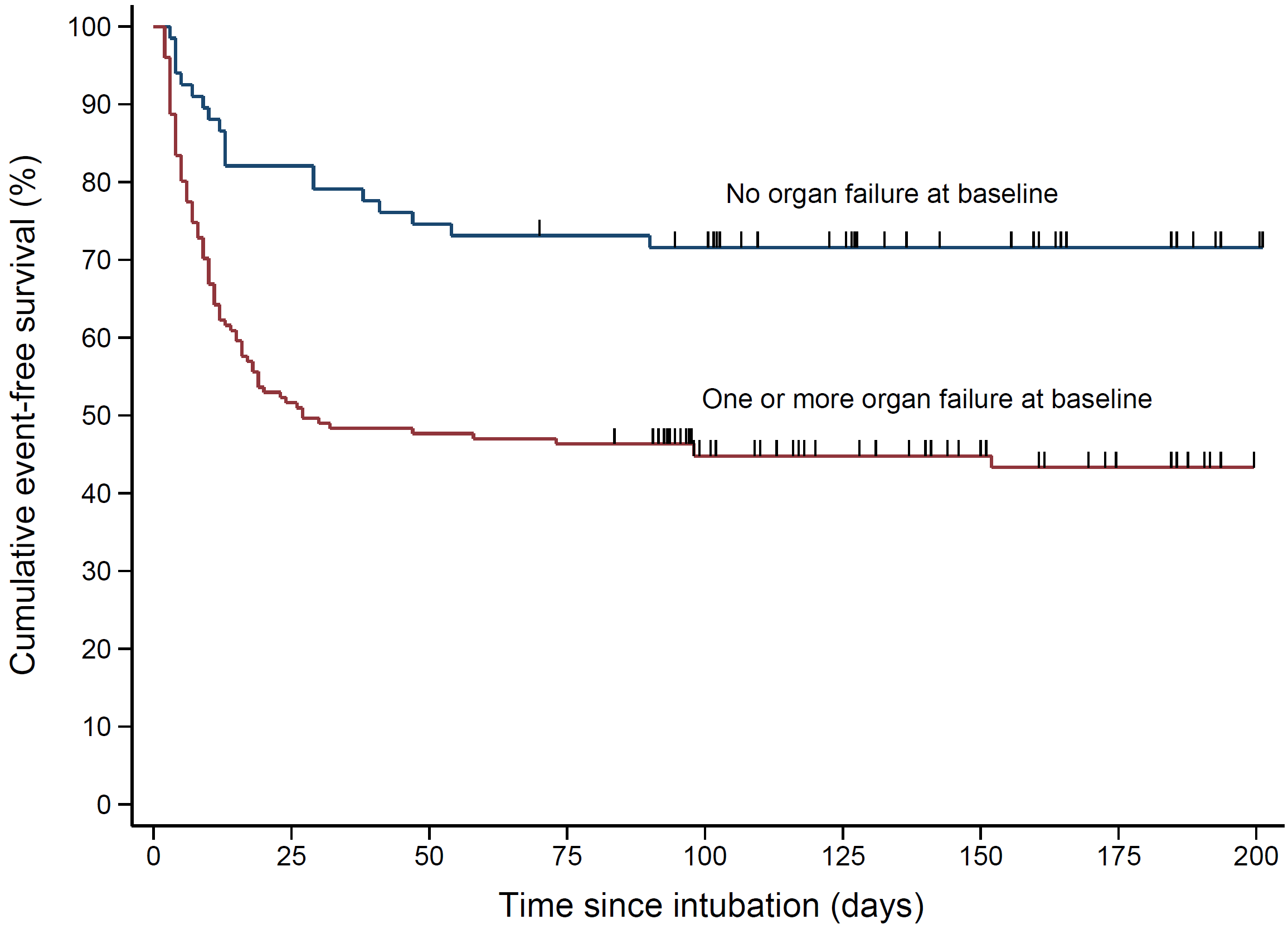

Supplement: S2 Fig — Hash marks on the plot indicate censoring. In two patients follow-up was censored at 70 days and 83 days. (TIF) [file pone.0135336.s002.tif]

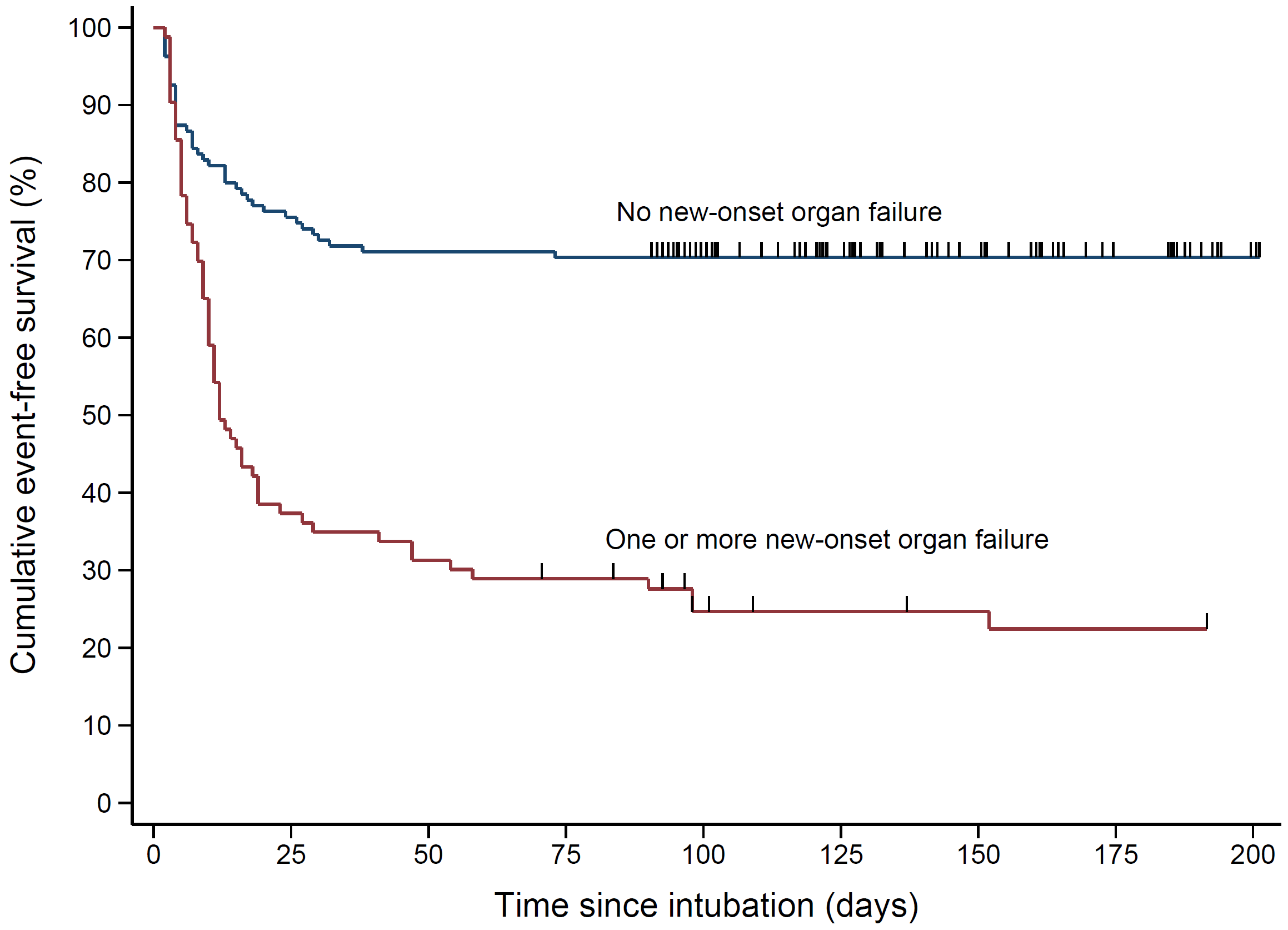

Supplement: S3 Fig — Hash marks on the plot indicate censoring. In two patients follow-up was censored at 70 days and 83 days. (TIF) [file pone.0135336.s003.tif]
