# Supplementary material for: Case-Mix, Care Processes, and Outcomes in Medically-Ill Patients Receiving Mechanical Ventilation in a Low-Resource Setting from Southern India: A Prospective Clinical Case Series
Source: PLoS One. 2015 Aug 11;10(8):e0135336. doi: 10.1371/journal.pone.0135336 (PMC4532502; doi:10.1371/journal.pone.0135336)
Supplement: S1 Table — APACHE II = Acute physiology and chronic health evaluation II. (DOCX) [file pone.0135336.s005.docx]

**S1 Table. Case details of mechanically ventilated patients that died after hospital discharge.**

| **No.** | **Age** | **Gender** | **Diagnosis** | **APACHE II score** | **VAP** | **Tracheostomy in situ** | **Duration of ventilation** | **Time of death^*^** |
| --- | --- | --- | --- | --- | --- | --- | --- | --- |
| 1. | 25 years | Female | Microscopic polyangiitis; diffuse alveolar hemorrhage | 23 | Yes | No | 14 days | 152 days |
| 2. | 20 years | Male | Tubercular meningitis | 13 | Yes | Yes | 32 days | 54 days |
| 3. | 29 years | Male | Hanging; hypoxic ischemic encephalopathy | 12 | Yes | No | 8 days | 98 days |
| 4. | 75 years | Male | Pneumonia | 15 | No | No | 9 days | 20 days |
| 5. | 21 years | Female | Hanging; hypoxic ischemic encephalopathy | 23 | Yes | Yes | 17 days | 58 days |
| 6. | 31 years | Female | Pneumonia | 11 | Yes | No | 10 days | 18 days |
| 7. | 55 years | Female | Hemotoxic snake bite; acute renal failure | 28 | Yes | No | 11 days | 13 days |
| 8. | 55 years | Male | Pneumonia | 25 | Yes | Yes | 17 days | 29 days |
| 9. | 31 years | Male | Tubercular meningitis | 12 | Yes | Yes | 20 days | 98 days |
| 10. | 60 years | Female | Obstructive sleep apnoea syndrome; type 2 respiratory failure | 28 | Yes | Yes | 21 days | 30 days |
| 11. | 75 years | Male | Sepsis; urinary tract infection | 34 | Yes | No | 14 days | 32 days |
| 12. | 52 years | Female | Pneumonia | 25 | No | No | 9 days | 26 days |
| 13. | 70 years | Male | Tetanus | 18 | Yes | Yes | 44 days | 73 days |
| 14. | 21 years | Female | Acute disseminated encephalomyelitis | 8 | Yes | Yes | 15 days | 38 days |
| 15. | 60 years | Male | Chronic kidney disease; pulmonary edema | 26 | No | No | 8 days | 24 days |
| 16. | 57 years | Male | Tetanus | 4 | Yes | Yes | 44 days | 90 days |

APACHE II = Acute physiology and chronic health evaluation II; VAP = ventilator-associated pneumonia

^*^Days since intubation
